# Anti-gallbladder cancer activities and toxicity studies of glycyrrhetinic acid derivative as a novel PPARγ agonist

**DOI:** 10.3389/fimmu.2025.1704994

**Published:** 2025-12-01

**Authors:** Min Liu, Haizhang Ma, Gaozhong Xiong, Mei Qi, Yuan Xia, Yunxue Zhao, Chunyan Ji, Juan Sun, Tao Sun

**Affiliations:** 1 Department of Pharmacy, Qilu Hospital of Shandong University, Jinan, Shandong, China; 2 Department of Hematology, Qilu Hospital, Cheeloo College of Medicine, Shandong University, Jinan, Shandong, China; 3 Department of Organ Transplant, Qilu Hospital of Shandong University, Jinan, Shandong, China; 4 Cheeloo College of Medicine, Shandong University, Jinan, Shandong, China; 5 Department of Pathology, Qilu Hospital of Shandong University, Jinan, Shandong, China; 6 School of Biological and Chemical Engineering, Zhejiang University of Science and Technology, Hangzhou, China

**Keywords:** glycyrrhetic acid, gallbladder cancer, PPARγ agonist, antitumor, toxicity

## Abstract

**Introduction:**

Chemotherapy remains the mainstay treatment for gallbladder cancer; however, its therapeutic efficacy is limited by poor chemosensitivity. Therefore, identifying more effective treatment strategies is essential for improving patient prognosis. In this study, we evaluated the antitumor activity of a novel PPARγ agonist, PG-4c, in gallbladder cancer and assessed its in vivo toxicity.

**Methods:**

Human gallbladder cancer cells were treated with PG-4c to examine its effects on the cell cycle, apoptosis, invasion, migration, and intracellular apoptotic signaling. A xenograft tumor model was used to assess the antitumor efficacy of PG-4c in vivo. Toxicity associated with PG-4c was evaluated in both mice and zebrafish.

**Results:**

PG-4c exhibited stronger anticancer activity than the standard chemotherapeutic agent gemcitabine. Its antitumor mechanisms involved inducing cell-cycle arrest, apoptosis, and necrosis through elevated intracellular reactive oxygen species levels and activation of cleaved caspase-3. PG-4c also impaired actin assembly, thereby inhibiting migration and invasion. In vivo, PG-4c significantly suppressed tumor growth in both zebrafish and mouse xenograft models. Notably, PG-4c demonstrated lower toxicity than the traditional PPARγ agonist pioglitazone, as supported by animal toxicity assays.

**Discussion:**

Our findings suggest that PG-4c holds strong potential as an effective chemotherapeutic candidate for gallbladder cancer. Given that PPARγ agonists are clinically approved drugs for dyslipidemia and diabetes, this class of agents may represent a promising new therapeutic approach for gallbladder cancer management.

## Highlights

- Anticancer efficacy of a novel PPARγ agonists based on the glycyrrhetic acid scaffod (PG-4c) was evaluated in gallbladder cancer *in vivo* and *vitro*.
- Comparison toxicity of PG-4c and traditional PPARγ agonist.
- PG-4c could be a new PPARγ lead-candidate agonist and potentially used for gallbladder cancer treatment.
- The introduction of methylpiperazine group at the C30 site can significantly improve the cytotoxicity.

## 1 Introduction

Gallbladder cancer (GBC) is a highly aggressive malignancy in the biliary tract system, often proving lethal due to early metastasis and delayed diagnosis, with a 5-year survival rate below 5% (1). While complete surgical resection remains the most effective therapeutic intervention, it seldom constitutes a radical cure, primarily due to the late-stage detection of the disease (2). Current treatment modalities, utilizing mitomycin C, 5-fluorouracil, gemcitabine, and platinum analogues, yield response rates ranging from 10% to 24% (3). Therefore, there is an imperative need to explore more efficacious treatment strategies to enhance the prognosis of GBC.

Peroxisome proliferator-activated receptor γ (PPARγ), a member of the nuclear receptor superfamily, plays a crucial role in diverse physiological and pathological processes. Activation of PPAR*γ* is linked to antiproliferative, pro-apoptotic, pro-differentiation, anti-inflammatory, and anti-metastatic effects in a variety of cancer cell lines and rodent carcinogenesis models (4). Recent evidence has also highlighted that peroxisome proliferator-activated receptors (PPARs) play a central role in regulating bile acid metabolism and maintaining hepatobiliary homeostasis, suggesting that dysregulation of PPAR signaling may contribute to biliary tract pathogenesis (5). Furthermore, PPARγ expression has been identified in gallbladder cancer, and its occurrence is intricately linked to the PPAR*γ* target (6).

Presently available PPAR agonists on the market manifest several side effects, and overactivation of PPAR*γ* may result in adverse outcomes such as elevated liver transaminase activity, mild edema, and anemia (7, 8). Therefore, the pursuit of compounds exhibiting potent PPARγ stimulation with minimal side effects is critical for GBC treatment. The emerging trend in current research emphasizes partial PPARγ activation, which theoretically balances high effectiveness with lower toxicity. In a prior study, a series of glycyrrhetinic acid derivatives targeting PPAR*γ* were designed and synthesized using computer-simulated drug design technology (7). Subsequent experimental results highlighted the potent antitumor activities of certain compounds, with PG-4c identified as a high-efficiency, low-toxicity lead compound. Building upon these promising results, we reported the antitumor efficacy of PG-4c, presenting it as a novel candidate PPAR*γ* agonist in a human GBC-SD line. Additionally, xenograft tumor growth inhibition rates of PG-4c and its toxicity were measured in zebrafish and mice, elucidating its potential as a drug for GBC treatment.

## 2 Experimental

### 2.1 Materials

The Bruker DPX400 spectrometer was utilized to acquire all ^1^HNMR, ^13^CNMR, FT-IR, and UPLC-MS spectra data. Thin layer chromatography employed a silica gel plate (SilicaGel60GF254), observed under ultraviolet light at wavelengths of 254 nm and 365 nm. The melting point was determined using a digital melting point instrument (Shen Guang, Shanghai, China). The FITC-labeled AnnexinV/PI apoptosis detection kit was sourced from Invitrogen-Life Technologies. Additionally, (2,7-Dichlorodihydrogen fluorescent diacetic acid DCFH-DA) was purchased from Beyotime Biotechnology Institute, TRITC-conjugated phalloidin was obtained from YEASEN, and DAPI from SparkJade (Qingdao, China). Antibodies, including Cyclin A Rabbit monoclonal antibody, Cyclin B1 Rabbit monoclonal antibody, Bcl-2 Rabbit monoclonal antibody, GAPDH Rabbit monoclonal antibody, beta-Actin Rabbit monoclonal antibody, and cleaved caspase-3 rabbit mAb, were purchased from Cell Signaling Technology (MA, US).

### 2.2 General procedure for PG-4c synthesis

The synthetic pathway for the target compound PG-4c closely aligns with a previously documented procedure (7) (**Scheme 1**). Initially, the carboxyl group of glycyrrhetinic acid underwent esterification for protection. Subsequently, the hydroxyl group at C3 was brominated and reacted with methylpiperazine to yield the target product.

**Scheme 1**. General procedure for the synthesis of PG-4c.

### 2.3 Aqueous solubility and lipid-water partition coefficient determination

To assess the drug potential of PG-4c preliminary to *in vivo* experiments, we conducted evaluations of its aqueous solubility and lipid-water partition coefficient. The determination of water solubility and fat-water partition coefficient employed the shaker method. Pure water or normal saline (1 mL) was introduced into the test vial, along with an excess of the compounds to be tested. The system was maintained at a constant temperature and subjected to agitation at 37°C for 24 h to achieve full saturation. After allowing for sedimentation, the supernatant was collected for concentration determination of the compounds in water through high-performance liquid chromatography. For the lipid/water partition coefficient assessment, noctanol served as the organic phase. A mixture of 1 mL pure water and 1 mL noctanol underwent constant temperature oscillation for 24 h to achieve full saturation of the two phases. Subsequently, 1 mg of the compound under investigation was introduced, and constant temperature oscillation continued for an additional 24 h.

### 2.4 Anti-tumor proliferation activity test

The human gallbladder cancer cell line GBC-SD cells were procured from the Chinese Academy of Sciences Cell Bank (Shanghai, China) and cultivated in RPMI1640 medium supplemented with 10% fetal bovine serum. Cells were seeded at a density of 3 × 10^3^ cells per well and subjected to treatment with varying concentrations of PG-4c for 2 days. Cell viability was assessed by measuring absorbance at 450 nm following the addition of CCK8.

### 2.5 Morphological observation

Cells were seeded in 6-well plates overnight and then exposed to PG-4c (0, 2, 5, and 10 μM) for 48 h. Images were then captured using a microscope equipped with a digital camera (Olympus).

### 2.6 Cell cycle analysis

GBC-SD cells were plated, and treated with PG-4c. The collected trypsin-digested cells were washed twice with PBS and fixed with 70% cold ethanol at -20°C overnight. Following PBS rinsing, 20 *μ*L RNase A was added and the cells were incubated at 37°C for 30 min. Subsequently, the treated cells were incubated with 400 *μ*L PI. Cell DNA content was measured using a flow cytometer (Beckman, USA), and the percentage of cells at each phase of the cell cycle was analyzed.

### 2.7 Analysis of cell apoptosis

Cells were plated in a 6-well plate at a density of 3×10^4^ cells/well, and cultured overnight. The culture medium was then replaced with fresh medium containing PG-4c at final concentrations of 0, 2, 5, and 10 *μ*M, and incubated for 48 h. After treatment, cells were trypsinized, washed with PBS, and centrifuged at 2000 rpm for 5 min. The resulting pellet was resuspended in 300 *μ*L staining solution (comprising 5 *μ*L AnnexinV-FITC and 10 *μ*L PI in Binding Buffer), gently mixed, and incubated for 15 min at room temperature in the dark. Analysis of the samples was then conducted using a FACSCalibur flow cytometer (Beckman, USA).

### 2.8 Wound healing assay

A scratch was introduced to the GBC-SD cell monolayer cultured in 6-well plates using a 200 μL plastic pipette tip. The GBC-SD cells were then treated with PG-4c at concentrations ranging from 0 to 10 μM. Microscopic images were acquired at 0, 24, and 36 h.

### 2.9 Cell migration and invasion assay

The assessment of tumor cell migration and invasion utilized the Transwell system (BD Biosciences, San Jose, CA, USA). GBC-SD cells were seeded into Matrigel-coated or uncoated chambers at a density of 5×10^4^ cells/well, and treated with PG-4c (5 and 10 μM). Simultaneously, complete medium was added to the lower chambers. Following a 24-h incubation period, non-penetrating cells were removed from the upper chambers using a cotton swab. Cells on the lower side were fixed with 4% paraformaldehyde for 20 min and then stained with crystal violet. The stained cells were then counted under a microscope.

### 2.10 Immunofluorescence staining and confocal microscopic analysis of actin filaments

Cells were cultured on cover slips and incubated with varying concentrations (2, 5, and 10 μM) of PG-4c. Subsequently, cells were washed with PBS, fixed with 4% paraformaldehyde, permeabilized with 0.1% Triton X-100, and then blocked with 1% BSA. Following another PBS wash, cells were incubated overnight with TRITC-conjugated phalloidin (1:500, Cytoskeleton, Inc.). On the next day, DNA was counterstained with DAPI (2 mg/mL) for 15 min at room temperature. After a final PBS wash, cells were observed under a Confocal Laser Scanning Microscope (100×, oil).

### 2.11 Measurement of intracellular reactive oxygen species production

The quantification of ROS was conducted through the 2,7-dichlorodihydrofluorescent diacetate (DCFH-DA) assay, following established protocols (9, 10). GBC-SD cells were seeded into 6-well plates and subjected to various treatments upon recovery. After 48 h of treatment, the cells were washed three times with PBS and treated with 10 μM DCFH-DA. Then, the cells were washed, trypsinized, and centrifuged, and the detection of cells in each sample was performed using flow cytometry.

### 2.12 Transcriptome sample preparation for sequencing

Total RNA was extracted from cells with or without 10 μM PG-4c treatment. Sequencing procedures were conducted by Novogene Corporation (Tianjin, China), encompassing the construction of cDNA libraries and sequencing via the Illumina HiSeq™ 2000 platform to yield expression libraries with a 50-nt read length. Preceding data analysis, the quality of raw sequencing reads was checked using the FastQC tool (v0.10.0). The sequencing data for each group were aggregated for direct comparison of RNA transcriptomes. Transcript expression levels were calculated using TPM (Transcripts Per Kilobase Million). Genes meeting the criteria of RPKM (Reads Per Kilobase per Million mapped reads) value≥20, fold change≥2, and DESeq P-adj value < 0.05 were considered to exhibit significant differences in expression between groups. Following data pre-processing, differential gene analysis was conducted between the PG-4c treatment group and the control group. Subsequently, Gene Set Enrichment Analysis was performed using the GSEA software.

### 2.13 Western blot analysis

Protein extracts (20 mg) prepared using RIPA lysis buffer were separated on 10% or 12% SDS-polyacrylamide gels and then transferred to PVDF membranes. Following a blocking step with 5% nonfat milk, the membranes were subjected to overnight incubation at 4 °C with specific antibodies targeting Cyclin A, Cyclin B1, PPAR*γ*, Bcl-2, cleaved caspase-3, GAPDH, or *β*-actin. Then, the membranes were washed and exposed to HRP-conjugated secondary antibodies at room temperature for 1h. Detection of protein bands was conducted using an ECL-Plus system as per the manufacturer’s instructions.

### 2.14 Evaluation of xenograft tumor growth inhibition by PG-4c

The gallbladder cancer xenograft model was established in nude mice and zebrafish. Six-week-old nude mice were obtained from Beijing Vital River Laboratory Animal Technology Co, Ltd. Human gallbladder cancer cells in logarithmic growth phase were cultured, digested, and adjusted to a concentration of 1×10^8^ cells/mL. Each nude mouse received a subcutaneous inoculation on the back with 1×10^7^ cells. Treatment initiation occurred in the second week, with mice receiving intraperitoneal injections of gemcitabine or PG-4c (40 mg/kg) twice weekly for three weeks (11). Tumor dimensions were measured with calipers, and tumor volume was calculated using the formula (L×W^2^)/2 (L, length; W, width). Post-experiment, mice were sacrificed, and tumors and livers were collected for subsequent analyses. Zebrafish embryos were incubated at 28°C in a standard environment with E3 medium (Sigma). Human gallbladder carcinoma cells, labeled with thiazole orange dye, were washed with PBS at least five times and microinjected into 48hpf zebrafish embryos under anesthesia with 0.2 g/L tetracaine (MS222, Sigma). The injection parameters were controlled to ensure 300 cells per embryo. Following anesthesia and washing, the injected zebrafish were exposed to a serial dilution (0–10 µM) of PG-4c and incubated at 28°C for 1 h, and then transferred to an incubator at 35°C to facilitate co-growth of zebrafish and human tumor cells (12, 13).

### 2.15 Determination of *in vivo* toxicity of PG-4c

The toxicity assessment aimed to evaluate potential adverse effects of PG-4c using mice and zebrafish. Male Balb/c mice, sourced from Beijing Vital River Laboratory Animal Technology Co., Ltd, were selected for the acute toxic experiment. For *in vivo* toxicity analysis, pioglitazone served as a control, given its primary target being the PPAR-γ receptor. The mice received a single dose of DMSO, pioglitazone, or PG-4c (500 mg/kg). Subsequently, all animals had unrestricted access to food and water post-drug administration. After 24 h, euthanasia was conducted through cervical dislocation, and the liver, spleen, and kidneys were dissected for histopathological examination. Zebrafish embryos from specific lines, including Tübingen (ZIRC [Zebrafish International Resource Center]), Tg (runx1:nEGFP), and Tg (kdrl:mCherry-CAAX), were employed to assess PG-4c drug toxicity. Prior to observations and image acquisitions, embryos were washed, dechorionated, anaesthetized with 0.016% tricaine (ethyl 3-aminobenzoate methanesulfonate salt; Sigma-Aldrich), and treated with PG-4c (50 µM). All procedures were approved by the Institutional Care and Use Committee of Shandong University and adhered to the Guidelines for the Care and Use of Laboratory Animals published by the National Institutes of Health.

### 2.16 Molecular docking

The molecular docking analysis of the compound with the three-dimensional X-ray structure of PPARγ (PDB code: 2HFP) was conducted using Discovery Studio (2019 edition) and the graphical user interface DS-CDOCKER protocol. The three-dimensional structure of PPARγ (2HFP) utilized in the docking study was obtained from the Protein Data Bank website. Additionally, the three-dimensional structure of PG-4c was constructed through chemical methods, and both structures underwent energetic minimization employing the MMFF94 force field and a minimum RMS gradient of 0.10. The elimination of bound waters on the protein structure of PPARγ and the addition of polar hydrogen were integral steps in the preparation process.

### 2.17 Statistical analysis

Data are expressed as mean ± standard deviation. Analyses of different treatment groups were conducted using analysis of variance (ANOVA) and Student t test with the Prism software (GraphPad Software, Inc.). All experiments were performed in triplicate, and significance was accepted at levels of p < 0.05 (*), p < 0.01 (**), and p < 0.001 (***).

## 3 Results and discussion

### 3.1 Chemistry, molecular docking study, aqueous solubility, and lipid-water partition coefficient of PG-4c

**Figure 1:**
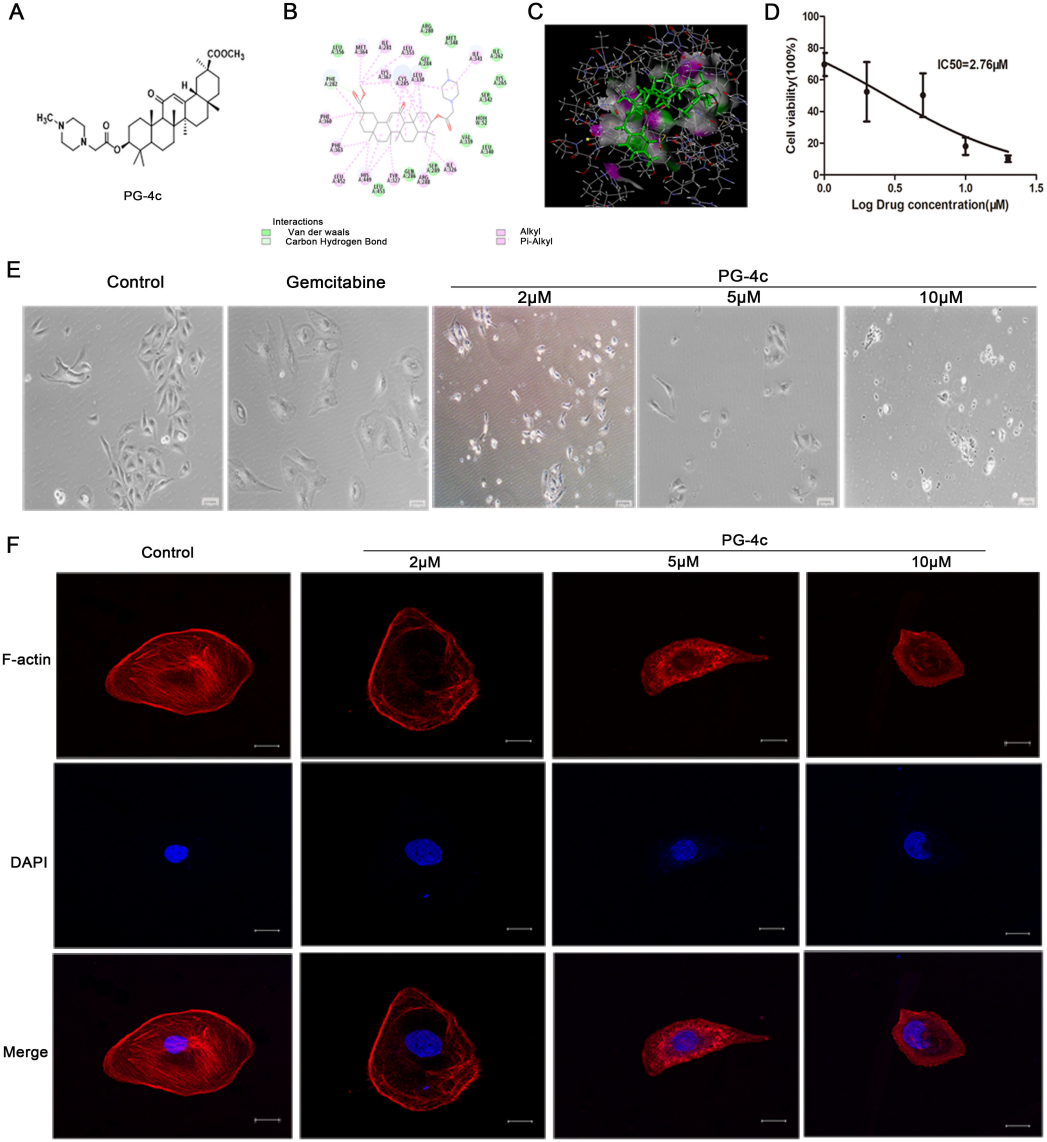
PG-4c inhibits cell proliferation and induces actin depolymerization in GBC-SD cells. **(A)** Glycyrrhetinic acid derivative 4c (PG-4c) docking model. **(B)** 2D docking diagram and **(C)** 3D docking diagram of PG-4c. **(D)** Cell viability rate, expressed as a percentage of the control, determined using the CCK-8 assay. **(E)** Morphological changes in GBC-SD cells induced by PG-4c photographed at 100× magnification. Scale bar, 100 *μ*m. **(F)** Confocal microscopy analysis of F-actin. Immunofluorescence staining of TRITC–Phalloidin (red) and DAPI (blue) in untreated and PG-4c-treated GBC-SD cells. Each group was assessed in triplicate, and representative data are shown. Original magnification, 630×. Scale bar, 20 μm.

The synthesis of PG-4c adhered to the outlined procedure in **Scheme 1**, with spectral data for the newly synthesized PG-4c detailed in the supplementary information. The molecular structure of compound PG-4c is depicted in **Figure 1A**, and the molecular docking results of PG-4c with PPAR*γ* are illustrated in **Figures 1B, C**. Upon analysis of the docking results, we observed that PG-4c effectively bound to the active site of PPAR*γ*, engaging with multiple amino acid residues and enhancing its affinity for the receptor. Specifically, PG-4c formed a hydrogen bond with Phe282 in the PPAR*γ* site. The cyclic structures demonstrated interactions with PPARγ amino acids through mechanisms such as van der Waals forces, alkyl, and Pi-alkyl interactions. Notably, our designed target compound exhibited characteristics consistent with a partial PPARγ activator, contributing to the heightened efficacy and reduced toxicity observed in PG-4c compared to its parent compound. In assessing the solubility of compound 4c, the thermodynamic equilibrium solubility in water was determined to be 0.281 mg/mL and in normal saline was 0.225 mg/mL. However, the hydrochloride form exhibited a substantial increase in solubility, with values of 7.75 mg/mL in water and 4.02 mg/mL in normal saline. These results underscore the favorable oral administration potential of PG-4c. Furthermore, the experimental LogP of PG-4c, at 1.25, adheres to the Lipinski Rule of Five, supporting its pharmacokinetic suitability.

### 3.2 PG-4c inhibits cell proliferation and induces actin depolymerization in GBC-SD cells

We conducted an *in vitro* assessment to evaluate the antiproliferative impact of PG-4c on GBC-SD cell lines. As depicted in **Figure 1D**, PG-4c exhibited notable inhibitory activity against tumor cell proliferation in GBC-SD cancer cells, with an IC_50_ of 2.76 *μ*M. The influence of PG-4c on cell morphology, a key indicator of cellular behavior, was documented through images captured after 48 h of treatment. Notably, PG-4c-treated cells exhibited a rounded and shrunken appearance (**Figure 1E**), signifying a cytotoxic effect on GBC-SD cells. Considering the pivotal role of actin in cell growth and shape maintenance, we sought to elucidate molecular events following PG-4c treatment. Visualization of the actin cytoskeleton in GBC-SD cells was accomplished through staining with TRITC-phalloidin, which selectively binds F-actin. As demonstrated in **Figure 1F**, PG-4c effectively disrupted intracellular stress fiber assembly in GBC-SD cells. Moreover, RNA sequencing was employed to validate the impact of PG-4c on GBC-SD cells comprehensively. GSEA analysis revealed a significant suppression of cell proliferation, disruption of actin assembly, and stress fiber formation in PG-4c-treated cells compared to the control group (**Supplementary Figures S1A, B**). These findings collectively underscore the multifaceted inhibitory effects of PG-4c on GBC-SD cell behavior at both morphological and molecular levels.

### 3.3 PG-4c induces cell cycle arrest and apoptosis of GBC-SD cells

**Figure 2:**
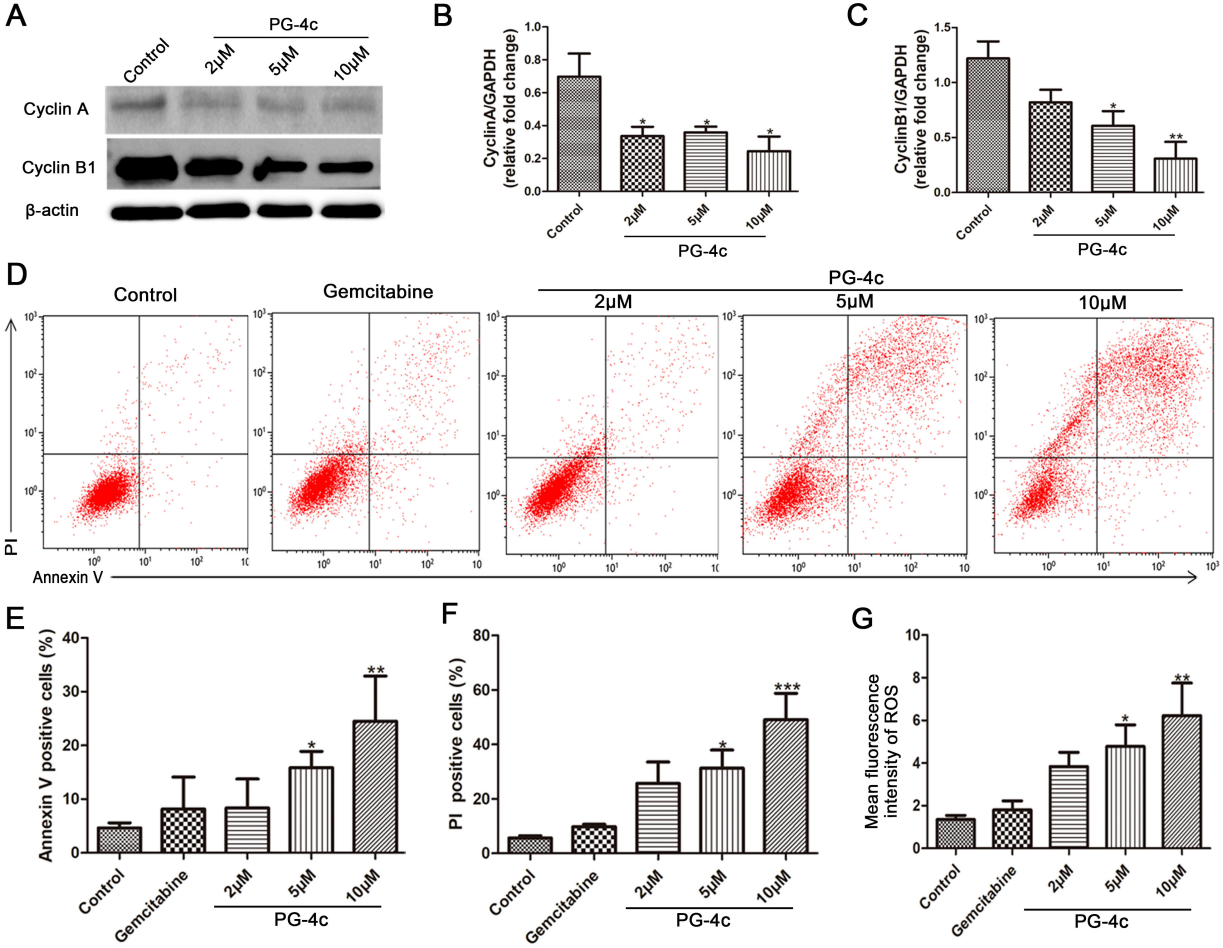
PG-4c induces cell cycle arrest and apoptosis of GBC-SD cells. **(A)** Effect of PG-4c treatment on the protein expression of cyclin A and cyclin B1 in GBC-SD cells. Experiments were performed in triplicate. **(B, C)** Levels of cyclin A and cyclin B1 quantified with Image J software, normalized to GAPDH (mean ± SEM, n = 3; *p < 0.05, **p < 0.01). **(D)** Representative flow cytometric analyses of GBC-SD cells stained with annexin V and PI. **(E)** Percentages of annexin V-positive cells with the above treatments. **(F)** Percentages of PI-positive cells. **(G)** Intracellular ROS level in GBC-SD cells treated with control, gemcitabine (20 *μ*M) and PG-4c (2, 5 and 10 *µ*M). Data are expressed as mean ± SEM; n = 3. *p < 0.05, **p < 0.01, ***p < 0.001 versus control.

The study progressed to investigate whether exposure to PG-4c, having previously demonstrated reduced viability of GBC-SD cells, could elicit cell cycle arrest. GBC-SD cells were treated with different concentrations (0, 2, 5, and 10 *μ*M) of PG-4c, and flow cytometric analysis of DNA content revealed an escalation in the G2/M phase population post-treatment (**Supplementary Figures S1C, D**). The groups treated with 5 and 10 *µ*M PG-4c exhibited significantly higher percentages of cells in G2/M phase (23.6% and 25.67%, respectively) compared to the control cells (17.76%). Conversely, the percentages of G0/G1 phase cells decreased to 68.35% and 64.51% with 5 and 10 *µ*M PG-4c, respectively (versus 75.67% in control cells). These results underscored the ability of PG-4c to induce dose-dependent cell cycle arrest at the G2/M phase in GBC-SD cells. Additionally, the protein expression of cyclins A and B1, implicated in the G2/M cell cycle phase, exhibited dose-dependent downregulation in response to PG-4c treatment, aligning with the observed cell cycle arrest (**Figures 2A–C**).

Furthermore, we evaluated the impact of PG-4c on apoptosis induction in GBC-SD cells, employing gemcitabine as a control. GBC-SD cells treated with PG-4c (2, 5, and 10 *µ*M) exhibited a notable increase in annexin V-positive cells, signifying heightened apoptosis compared to gemcitabine-treated cells (**Figures 2D–F**). Conversely, compared to the control (5.64%), the percentages of PI(+) GBC-SD cells significantly rose to 31.3% and 49.1% with 5 and 10 *µ*M PG-4c treatment for 48 h, respectively. Gemcitabine treatment resulted in 9.82% PI-positive cells. These results conclusively demonstrate the concentration-dependent induction of apoptosis by PG-4c in GBC-SD cells.

ROS, recognized as single-electron reduction products of oxygen *in vivo* (14), act as second messengers to upregulate proapoptotic protein expression. Therefore, the investigation of PG-4c’s effect on ROS levels in GBC-SD cells was undertaken. Results presented in **Figure 2G** illustrate a significant dose-dependent increase in ROS levels induced by PG-4c, supporting the previously described apoptosis data.

### 3.4 Treatment of GBC-SD cells with PG-4c upregulates PPARγ and triggers apoptosis signaling pathways

**Figure 3:**
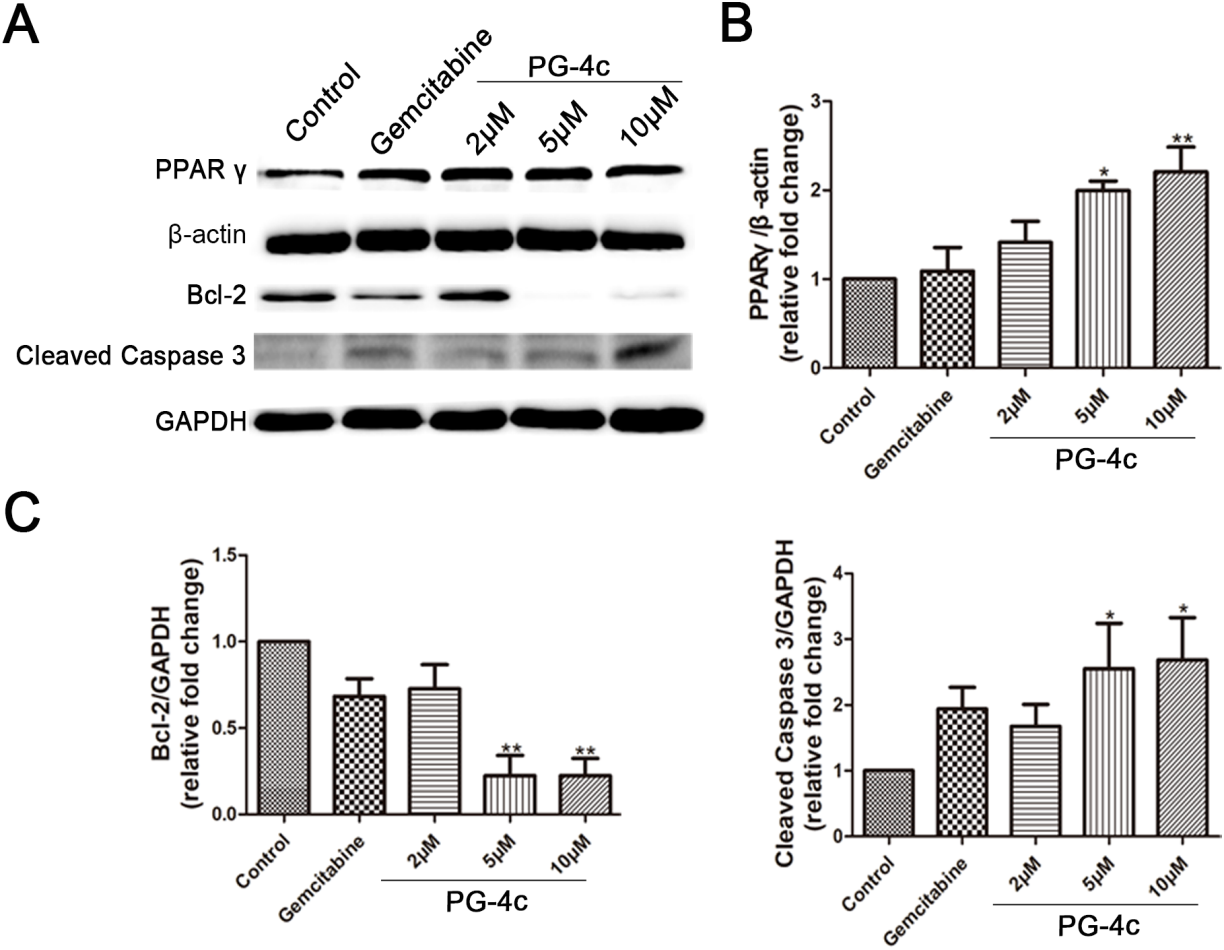
Validation of critical differentially expressed proteins involved in apoptosis regulation in PG-4c-treated GBC-SD cells. **(A)** Representative Western blotting analysis of PPAR*γ*, Bcl-2, and cleaved Caspase 3 expression in cultured GBC-SD cells. **(B, C)** The protein concentrations of PPAR*γ*, Bcl-2, and cleaved caspase quantified with Image J software (mean ± SD, n = 3; *p < 0.05, **P < 0.01).

RNA sequencing was employed to discern differentially expressed apoptotic signal transcripts between control and PG-4c-treated GBC-SD cells, as illustrated in **Supplementary Figure S1E**. Western blot analysis was then conducted to elucidate the targeted agonistic activity of PG-4c against PPAR*γ*, focusing on the Bcl-2 and cleaved caspase 3 signaling pathways. As depicted in **Figures 3A–C**, PG-4c induced an increase in PPAR*γ* content and altered the expression of proteins associated with apoptosis in cancer cells. This observation suggests that PG-4c may modulate the intracellular expression levels of pro-survival proteins by activating PPAR*γ*, thereby eliciting antitumor activity.

### 3.5 PG-4c prevents migration and invasion of GBC-SD cells

**Figure 4:**
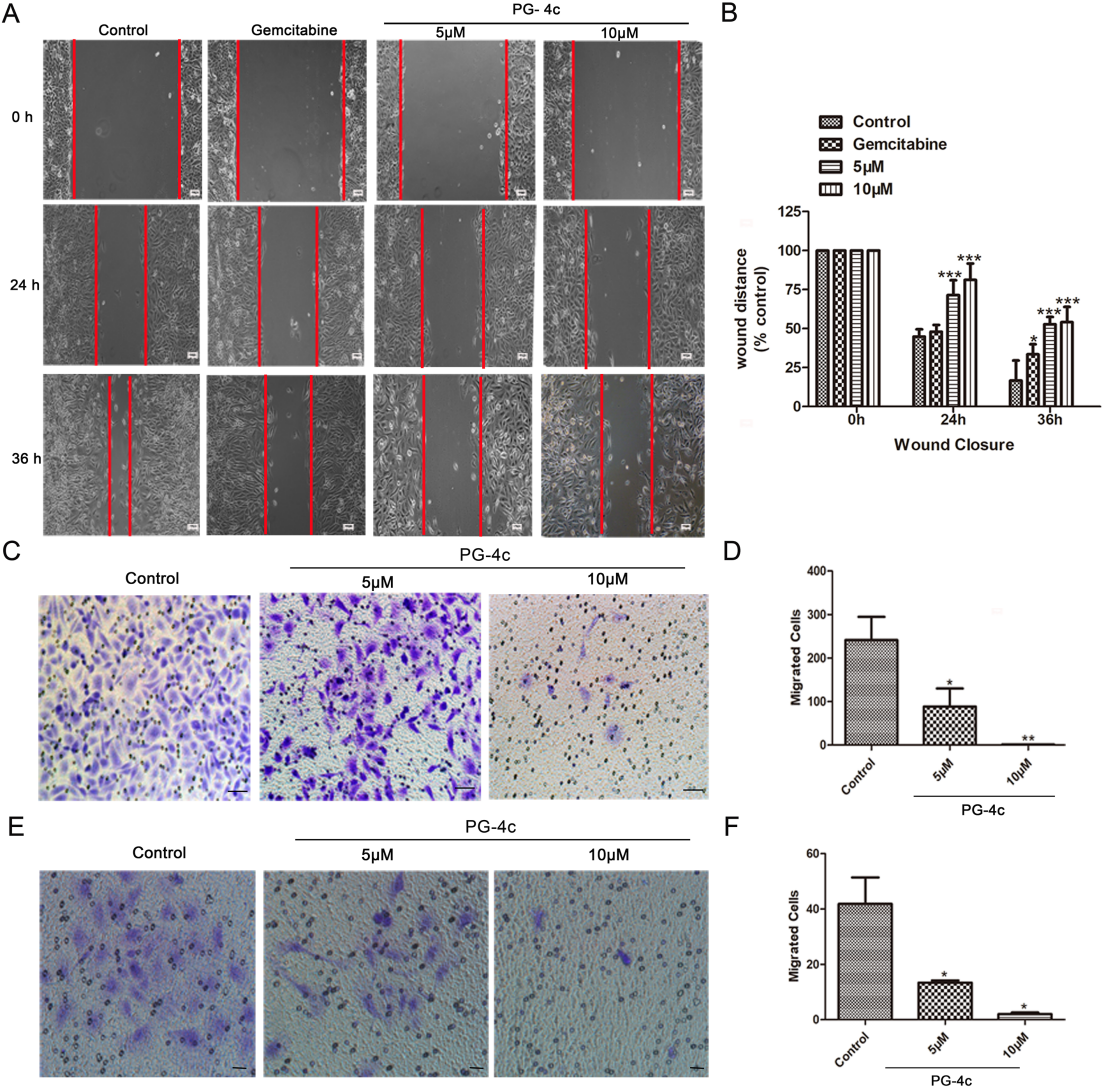
PG-4c restrains migration and invasion of GBC-SD cells. **(A, B)** GBC-SD cell migration assessed using wound-healing scratch assay. **(A)** Representative photomicrographs of gemcitabine (20 *μ*M)- and PG-4c-treated cells at 0, 24 and 36 (h) Red lines indicate migrating cell edges. **(B)** Bar graphs show wound closure in treated cells as a percentage of control cells. Results from three independent experiments are shown. *p < 0.05, **p < 0.01, ***p < 0.001 versus control. **(C)** Cellular migration analysed using a Boyden chamber assay. Representative fields were observed and photographed at 100× magnification; scale bar, 100 *μ*m. **(D)** Quantification of transwell migration assay of PG-4c-treated cells. Data are mean ± SEM; n = 3. *p < 0.05, **p < 0.01 versus control. **(E)** Representative images of transwell invasion assay. Invaded cells were photographed at 100× magnification. Scale bar, 100 *μ*m. **(F)** Quantification of invaded cells. Data are mean ± SEM; n = 3. *p < 0.05, **p < 0.01 versus control.

Migration and invasion are pivotal processes in cancer metastasis (15, 16). We sought to investigate the impact of PG-4c on these processes in GBC-SD cells. Scratch assays revealed a significant delay in wound closure at 24 and 36 h with PG-4c (5 and 10 μM) compared to untreated cells (**Figures 4A, B**). Furthermore, the Boyden chamber assay demonstrated a reduction in cell migration in GBC-SD cells upon PG-4c treatment, as depicted in **Figures 4C, D**. Next, we assessed the influence of PG-4c on tumor cell invasion. As shown in **Figures 4E, F**, the number of invasive GBC-SD cells markedly decreased after 24 h of PG-4c treatment compared to the negative control group. GSEA analysis further supported these findings, indicating that PG-4c has the capacity to impede cell migration (**Supplementary Figure S1F**). In summary, these results highlight the potent inhibitory effect of PG-4c on the migration and invasion of GBC-SD cells.

### 3.6 PG-4c inhibits growth of xenograft gallbladder carcinoma cells

**Figure 5:**
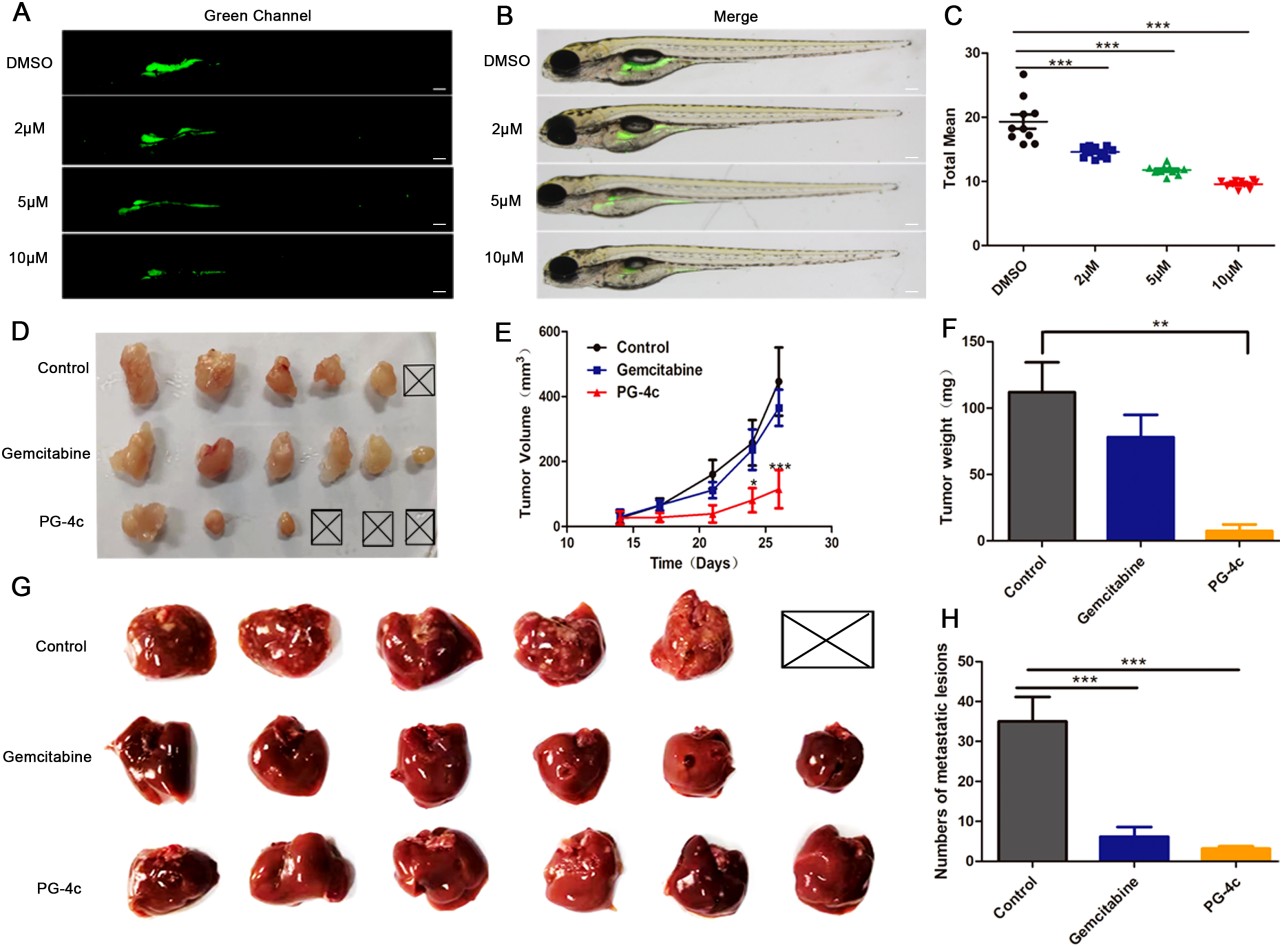
PG-4c inhibits growth of xenograft gallbladder carcinoma cells. **(A)** Green fluorescence expression of thiazole orange-labelled GBC-SD cell-transplanted zebrafish under different treatments. Scale bar, 100 *μ*m. **(B)** Superposition of fluorescence image and bright-field image of zebrafish. Scale bar, 100 *μ*m. **(C)** Quantification of fluorescence region in the figure above. Sample local mean, n=9. **(D)** Representative images of excised tumours from different experimental groups (n =6). **(E)** Tumour growth in different treatment groups (n =6). The graph shows tumour volume throughout treatment. Statistical significance was calculated using two-way ANOVA. **(F)** Statistical diagram of tumour weight (mean ± SD, n =6). **(G)** Images of the dissected liver (n=6/group). **(H)** Statistical diagram of liver metastatic lesions (mean ± SD, n=6), *p<0.05, **p<0.01, ***p<0.001.

We further investigated the therapeutic efficacy of PG-4c against gallbladder cancer *in vivo* using a xenograft carcinoma model. Thiazole orange-labeled GBC-SD cells were transplanted into the yolk sac of 2 days post-fertilization (dpf) zebrafish embryos. One day post-injection, tumor cells exhibited robust growth and stable proliferation. Following incubation with DMSO or PG-4c at concentrations of 2, 5, and 10 *μ*M for 48 h at 2 days post-injection (dpi), a significant reduction in the tumor area of zebrafish embryos treated with PG-4c was observed, as depicted in **Figures 5A, B**. This corresponded to tumor growth inhibition rates of 24.6%, 39.2%, and 60.4%, respectively (**Figure 5C**). Encouraged by these results, we proceeded to establish a nude mouse xenograft tumor model. Tumors became apparent on day 15 post-implantation, initiating drug treatment. After three weeks, tumors were excised and weighed, with no visible masses observed in three mice from the PG-4c group. Moreover, as illustrated in **Figures 5D, E**, PG-4c treatment significantly impeded gallbladder tumor growth, resulting in decreased tumor weight (**Figure 5F**). Notably, liver metastasis substantially decreased in mice treated with PG-4c compared to control mice (**Figures 5G, H**). These effects were more pronounced than those observed in the gemcitabine-treated group, indicating the strong inhibitory impact of PG-4c on gallbladder cancer.

### 3.7 PG-4c exhibits low toxicity

**Figure 6:**
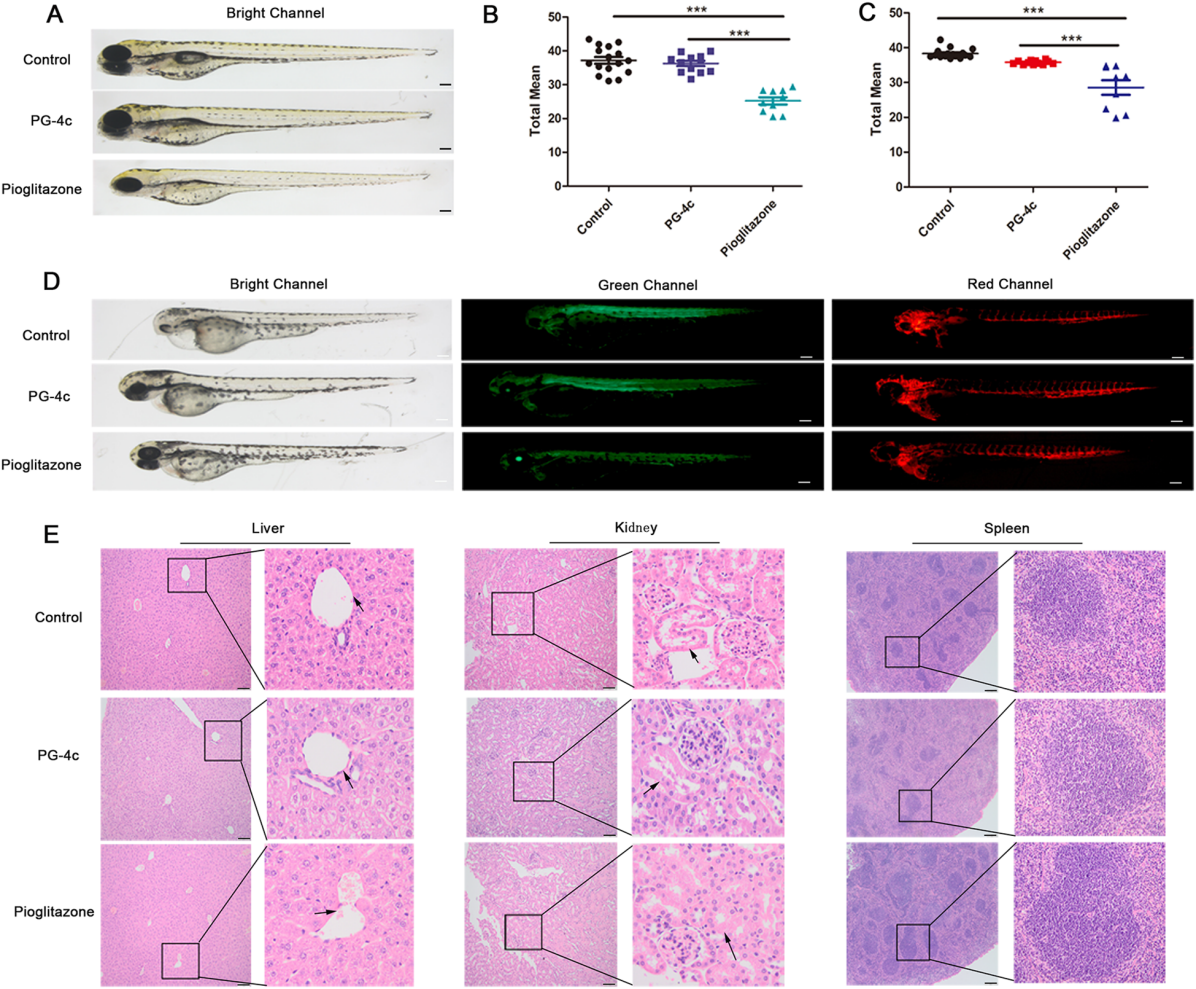
PG-4c exhibits low toxicity. **(A)** Zebrafish embryos treated with PG-4c show relatively full pigmentation compared to pioglitazone. Scale bar, 100 *μ*m. **(B)** and **(C)** show the mean detected expression levels of runx1P2:GFP and kdrl:mCherry. Scale bar, 100 *μ*m. Error bars represent mean ± SEM. Two-way ANOVA; *p < 0.05; **p < 0.01; ***p < 0.001. **(D)** Representative fluorescence microscopy image of 6 dpf runx1P2:GFP, kdrl:mCherry double transgenic embryos. **(E)** Liver, kidney and spleen histological sections from different groups. Liver and kidney images represent 100 *μ*m, and spleen images represent 200 *μ*m.

Next, we assessed the toxicity of PG-4c on normal cell proliferation and division using zebrafish, complemented by an examination of organ toxicity implicated in drug metabolism and clearance in mice. The *in vivo* animal toxicity assay conducted in zebrafish embryos demonstrated that PG-4c neither resulted in embryonic mortality nor induced teratogenic effects. Notably, pigmentation remained unaffected, unlike embryos exposed to pioglitazone. PG-4c-treated zebrafish embryos exhibited full pigmentation in both body and eyes (**Figure 6A**). Given the pivotal role of runx1 in blood development, particularly in hematopoietic stem cells (HSCs), we studied vascular and HSC development using endothelial and runx1-specific transgenic zebrafish line (flk1:egfp-runx1/kdrl:mCherry-CAAX) embryos. The results revealed delayed angiogenesis and compromised runx1 expression in pioglitazone-treated zebrafish embryos (**Figures 6B–D**). In the acute toxicity assessment in mice, hepatic tissues of PG-4c-treated mice displayed nearly normal architecture, while pioglitazone resulted in central vein congestion, irregular sinusoidal shape, and dilatation. Renal sections from PG-4c-treated mice exhibited mild tubular damage and hypertrophy of renal tubule epithelium, contrasting with pioglitazone-treated mice, where severe tubular damage and hypertrophy were evident. Examining the spleen, composed of white and red pulps, and surrounded by a connective tissue capsule, revealed a discernible increase in white pulps with morphological changes in the pioglitazone-treated group, whereas the PG-4c-treated group displayed no alterations in white pulps. These results underscore that PG-4c exhibits lower toxicity compared to pioglitazone (**Figure 6E**).

## 4 Conclusion

Exploring a more effective treatment strategy for GBC is imperative. Currently, diverse PPARγ-activating derivatives have been synthesized for human cancer treatment. For instance, PPAR*γ* ligands designed to impact cell differentiation, proliferation, and apoptosis have recently been developed (17). In our prior research, we targeted PPAR*γ* in two human cancer cell lines (MCF-7 and HepG2) (**Supplementary Table 1**) (7). In this study, we employed PPAR*γ* as a drug target to investigate the anti-GBC-SD activity of PG-4c. The results revealed high efficiency of PG-4c in GBC-SD cell lines with low *in vivo* toxicity. This may be attributed to the fact that, aside from interacting with amino acid Phe 282, the binding of PG-4c to the active site of PPAR*γ* relies predominantly on weak forces rather than strong ones, such as hydrogen bonds. Moreover, recent studies have highlighted that partial PPARγ agonists can achieve balanced receptor activation and reduce the adverse effects commonly associated with full agonists, while retaining anticancer efficacy (18). In line with this concept, a newly synthesized series of glycyrrhetinic acid derivatives containing piperazine moieties were shown to exert strong antitumor activities through PPARγ and caspase-3-mediated pathways, further supporting our design rationale for PG-4c (19). These findings collectively reinforce the potential of PG-4c as a low-toxicity PPARγ-targeted therapeutic candidate for GBC.

In this study, we assessed the efficacy of PG-4c as a novel therapeutic agent for human GBC. PG-4c demonstrated a significant inhibitory effect on GBC-SD cell proliferation in our experiments. F-actin assembly was disrupted after PG-4c treatment. Furthermore, PG-4c induced G2/M cell cycle arrest, as evidenced by a decrease in the expression of cyclins A and B post-treatment. These findings hold significance as regulating the cell cycle is pivotal in addressing GBC-SD. Apoptosis, a natural defense mechanism against cancer, represents a valuable strategy for cancer treatment (20). Our results indicated that PG-4c induces apoptosis in GBC-SD cells. ROS are known to damage subcellular components, leading to cell death (21). The elevated ROS levels post-PG-4c treatment elucidate the observed toxicity in human GBC-SD cells. Numerous studies have established the involvement of ROS in apoptosis induced by diverse stressors (22, 23). Immunoblotting revealed a decrease in Bcl-2 expression and an increase in cleaved caspase-3 levels in PG-4c-treated GBC-SD cells compared to control cells. Beyond its pro-apoptotic effects, PG-4c markedly reduced the migratory and invasive capabilities of GBC-SD cells. This aligns with accumulating evidence that PPARγ activation can modulate cytoskeletal organization and suppress tumor cell motility (24, 25). Specifically, Jang et al. (24) demonstrated that the PPARγ ligand CDDO downregulated MMP-9 expression and inhibited breast cancer cell migration and invasion, highlighting the regulatory role of PPARγ signaling in metastatic behavior. Chi et al. (25) summarized that PPARγ modulators suppress tumor progression by inducing apoptosis and impairing epithelial–mesenchymal transition across multiple cancer types. These findings collectively support that PG-4c may exert similar PPARγ-dependent anti-metastatic effects in GBC.

The tumor growth inhibition rate of GBC-SD xenografts transplanted into zebrafish and nude mice, upon treatment with PG-4c, demonstrated a marked increase compared to gemcitabine. PG-4c exhibited significant anticancer activity while causing minimal damage to zebrafish embryos and mouse organs. These findings are consistent with recent studies showing that PPARγ modulators can suppress tumor growth *in vivo* with a favorable safety profile (25). Likewise, recent findings on glycyrrhetinic acid derivatives highlight their improved *in vivo* antitumor efficacy following structural refinement (19), in agreement with the potent yet well-tolerated effects of PG-4c seen in our zebrafish and mouse models. Similar results have also been reported for clinically used PPARγ agonists such as pioglitazone, which reduced tumor growth and altered tumor metabolism in xenograft models (26). Moreover, loss-of-function studies suggest that stromal PPARγ activity plays a decisive role in determining the therapeutic window for these agents (27), which may help to explain the broad efficacy and low toxicity observed with PG-4c in our experiments. From a pharmacological perspective, ongoing research continues to refine glycyrrhetinic acid scaffolds to improve solubility, bioavailability, and overall safety (28), reinforcing the translational promise of PG-4c as a well-tolerated anticancer candidate.

Furthermore, our results showed that PG-4c markedly inhibited cell migration and tumor progression compared with gemcitabine, as evidenced by delayed wound closure *in vitro* and reduced tumor burden and liver metastasis *in vivo*. Previous evidence indicates that PPARγ signaling can influence chemosensitivity in biliary and pancreatic cancers. Asukai et al. reported that PPARγ-positive cholangiocarcinoma patients receiving adjuvant gemcitabine therapy had significantly longer disease-free survival than PPARγ-negative cases (29). Similarly, Jiang et al. demonstrated that activation of PPARγ by a black-phosphorus-based agonist overcame gemcitabine resistance in pancreatic adenocarcinoma by suppressing cancer stemness and downregulating resistance-related biomarkers (30). These findings suggest that the enhanced antitumor efficacy of PG-4c over gemcitabine may be related to its ability to activate PPARγ and mitigate resistance mechanisms associated with gemcitabine treatment.

**Figure 7:**
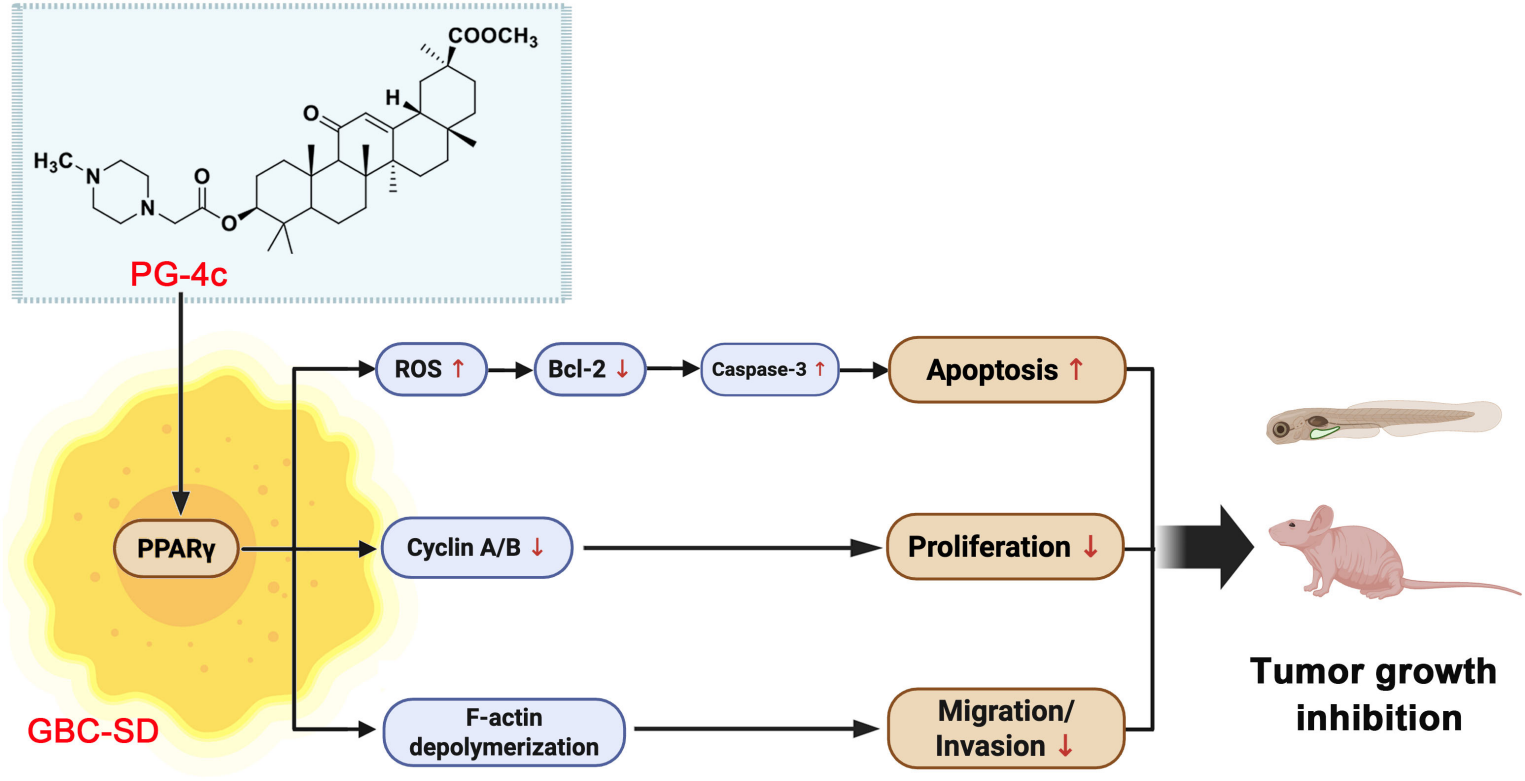
Schematic representation of the proposed anticancer mechanism of PG-4c in GBC.

Taken together, these results highlight PG-4c as a promising low-toxicity PPARγ-targeted compound for gallbladder cancer therapy. Still, the precise mechanism underlying its selective efficacy warrants further study. One intriguing possibility is that the upregulation of PPARγ observed after PG-4c exposure reflects a feedback activation or stabilization effect on the receptor itself. Another is that the unique interaction pattern of PG-4c—dominated by weak non-covalent forces with residues such as Phe282—contributes to its partial agonist behavior and reduced systemic toxicity. This selective binding mode likely limits the full conformational activation of PPARγ and the recruitment of coactivators that drive systemic side effects observed with full agonists such as pioglitazone, including fluid retention and hepatotoxicity. This mechanistic difference may account for the favorable safety profile of PG-4c observed in our *in vivo* studies (**Figure 7**).

Future studies combining structure-based modeling, binding kinetics, and long-term *in vivo* evaluations will be essential to clarify these mechanisms. Such work will not only elucidate the biological basis of PG-4c’s activity but also guide the rational design of next-generation glycyrrhetinic acid–derived PPARγ agonists with improved therapeutic indices.

## Data Availability

The original contributions presented in the study are included in the article/**Supplementary Material**. Further inquiries can be directed to the corresponding authors.

## Supplementary material

Scheme 1 General procedure for PG-4c synthesis.

Supplementary Figure 1 Supporting analyses related to PG-4c–induced transcriptional and cell-cycle changes in GBC-SD cells. **(A)** Gene set enrichment analysis of regulation of cell population proliferation. **(B)** Gene set enrichment analysis of microtube cytoskeleton organization. **(C)** Cell cycle pattern of GBC-SD cells in different groups. **(D)** Statistical data of cell cycle distribution. *p < 0.05, **p < 0.01, ***p < 0.001 versus control. Data are presented as the mean ± SEM of three independent experiments. **(E)** Heatmap of the mRNA expression of critical differentially expressed genes using RNA-seq data derived from two samples (treated and untreated PG-4c, p < 0.05). (F) GSEA of migration-related gene sets indicating suppression of motility pathways following PG-4c treatment.
