# Supplementary material for: Anti-gallbladder cancer activities and toxicity studies of glycyrrhetinic acid derivative as a novel PPARγ agonist
Source: Front Immunol. 2025 Dec 1;16:1704994. doi: 10.3389/fimmu.2025.1704994 (PMC12702885; doi:10.3389/fimmu.2025.1704994)
Supplement: Scheme 1 — General procedure for PG-4c synthesis. [file Table1.docx]

**The ^1^H NMR and ^13^C NMR spectra data of synthesized 18*β*-glycyrrhetinic acid derivative 4c.**

**Methyl-2,4a,6a,6b,9,9,12a-heptamethyl-10-(2-(4-methylpiperazin-1-yl)acetoxy)-13-oxo-1,2,3,4,4a,5,6,6a,6b,7,8,8a,9,10,11,12,12a,12b,13,14b-icosahydropicene-2-carboxylate (4c)**


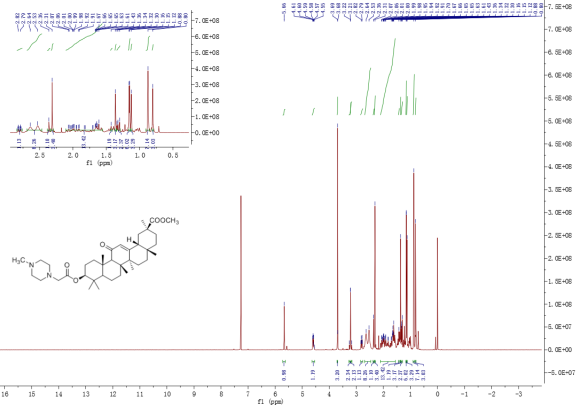

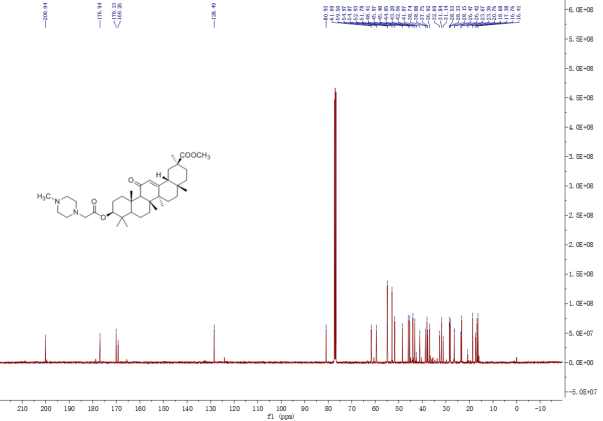


White powder, Mp: 264-267℃, Yield: 79%.^1^H NMR (400 MHz, CDCl_3_) δ 5.66 (s, 1H), 4.58 (dt, *J* = 17.6, 7.0 Hz, 1H), 3.69 (s, 3H), 3.28 – 3.16 (m, 2H), 3.21 (d, *J* = 2.6 Hz, 2H), 2.80 (dt, *J* = 13.4, 3.3 Hz, 1H), 2.58 (d, *J* = 43.9 Hz, 8H), 2.36 (s, 1H), 2.31 (s, 3H), 2.11 – 1.56 (m, 13H), 1.43 (s, 1H), 1.36 (s, 3H), 1.34 – 1.29 (m, 2H), 1.15 (d, *J* = 3.6 Hz, 6H), 1.12 (s, 3H), 0.88 (s, 7H), 0.80 (s, 3H). ^13^C NMR (101 MHz, CDCl_3_) δ 200.04, 176.94, 170.13, 169.26, 128.49, 80.93, 61.69, 59.50, 54.97, 54.87, 52.93, 51.78, 48.41, 45.97, 45.40, 44.05, 43.20, 42.56, 41.07, 38.74, 38.08, 37.75, 36.92, 32.69, 31.84, 31.14, 28.53, 28.33, 28.15, 26.47, 26.42, 23.67, 23.36, 20.76, 18.68, 17.38, 16.76, 16.41.

Supplement Table1. *In* *vitro* anticancer activities (IC _50_ *μ*M) against two human tumor cell lines

| Compounds | IC_50_±SD ( *μ*M) | |
| --- | --- | --- |
|  | MCF-7 | HepG2 |
| **4c** | 6.898±0.839 | 9.949±0.998 |
| **4e** | 31.588±1.500 | 21.939±1.341 |
| **4l** | 16.320±1.213 | 20.510±1.312 |
| **4m** | 18.003±1.255 | 34.163±1.534 |
| **4n** | 34.445±1.537 | 26.260±1.419 |
| **4o** | 11.572±1.063 | 21.040±1.302 |
| **4p** | 8.073±0.907 | 16.614±1.220 |
| **4q** | 9.500±0.978 | 25.585±1.408 |

Supplement Figure1 .The structures of relative compounds.

Supplement Figure2. FT-IR and UPLC-MS spectra of PG-4c


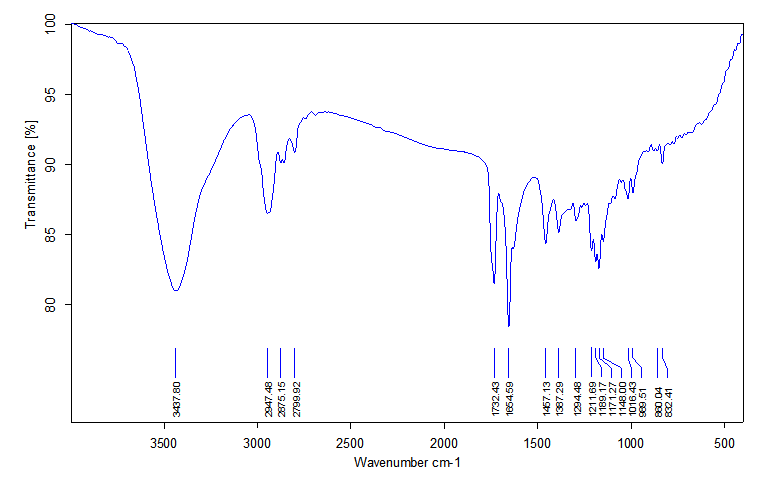

| (absorption peak) / cm^-1^ | absorption intensity | Vibration form | assignment |
| --- | --- | --- | --- |
| **（1）**3600-3400 | S | ν_N-H_ | Nitrogen-hydrogen stretching vibration |
| **（2）**2900-2700 | M | ν_C-H_ | Hydrocarbon stretching vibration of alkanes |
| **（3）**1800-1600 | S | ν_-C＝O-_ | Deformation vibration of carbonyl group |
| **（4）**1500-1400 | M | ν_-C＝C-_ | Deformation vibration of the carbon-carbon double bond |
| **（5）**1200-800 | W、M | ν_CH_、δ_CH2_ | Stretching vibration of methylene hydrocarbon |


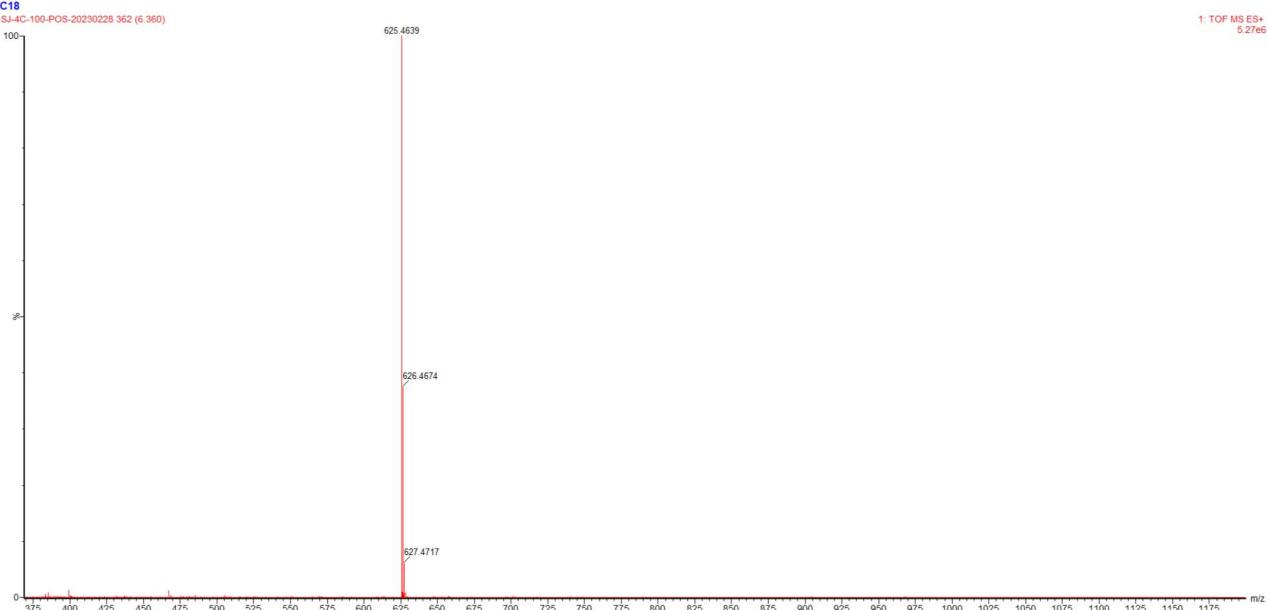


Supplement Figure 3. HPLC traces of PG-4c.


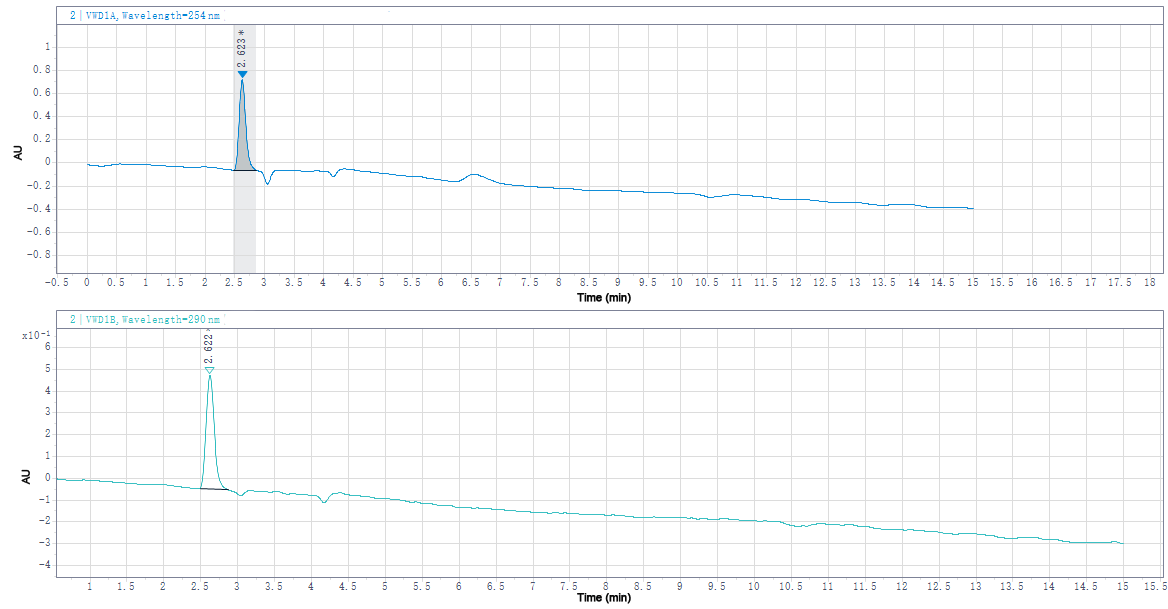


|  | Name | Retention  Time  (minutes) | Area  (μV*s) | %Area | Height  (μV) | The type of credit | Content | Unit | Peak type | Peak code |
| --- | --- | --- | --- | --- | --- | --- | --- | --- | --- | --- |
| 1 |  | 2.623 | 6.145 | 100 | 0.785 | BB |  |  | Î’Ö^a^ |  |
| 2 |  | 2.622 | 4.052 | 100 | 0.519 | BB |  |  | Î’Ö^a^ |  |
