# Supplementary figures and images for: Anti-gallbladder cancer activities and toxicity studies of glycyrrhetinic acid derivative as a novel PPARγ agonist
Source: Front Immunol. 2025 Dec 1;16:1704994. doi: 10.3389/fimmu.2025.1704994 (PMC12702885; doi:10.3389/fimmu.2025.1704994)

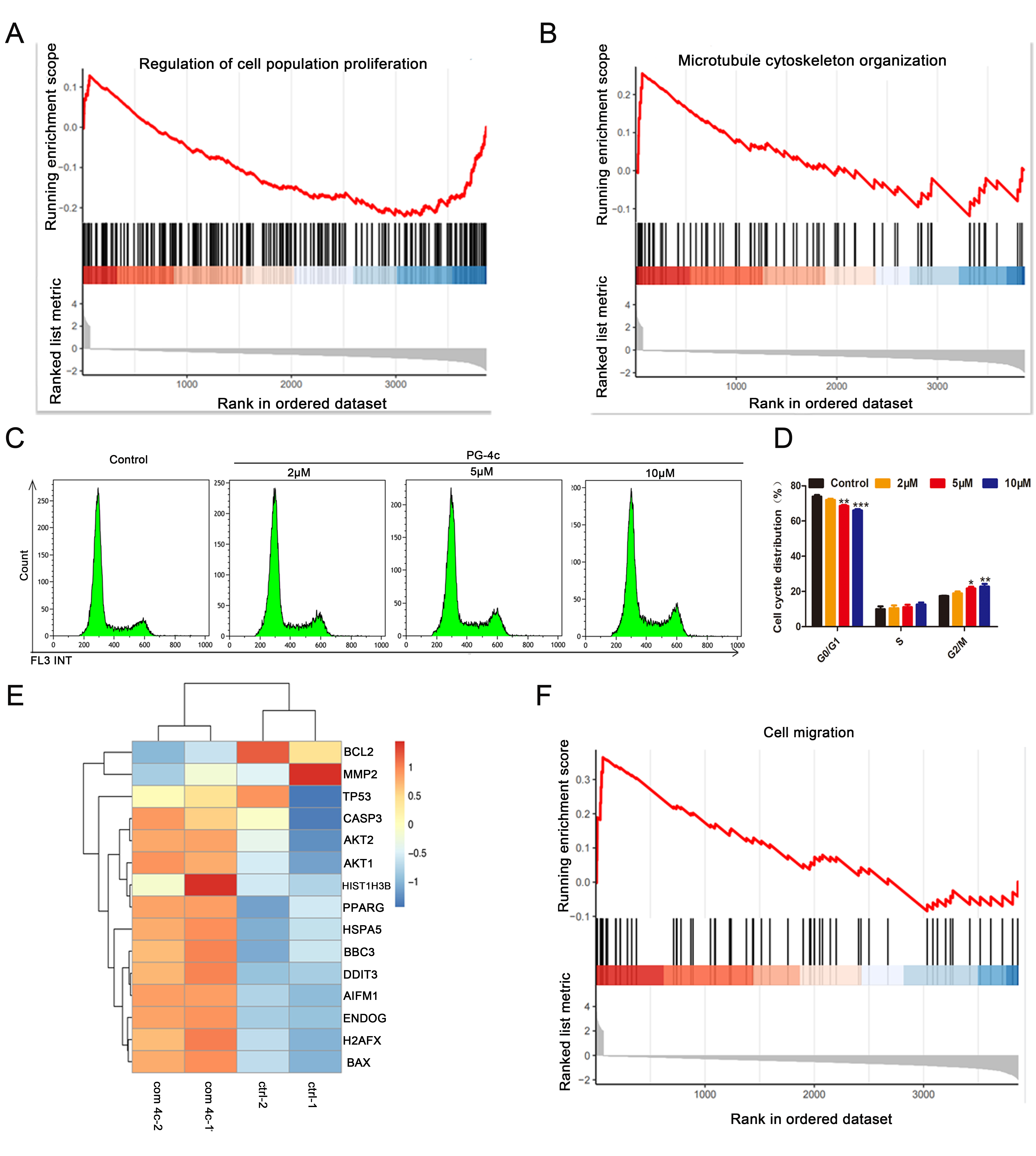

Supplement: Supplementary Figure 1 — Supporting analyses related to PG-4c–induced transcriptional and cell-cycle changes in GBC-SD cells. (A) Gene set enrichment analysis of regulation of cell population proliferation. (B) Gene set enrichment analysis of microtube cytoskeleton organization. (C) Cell cycle pattern of GBC-SD cells in different groups. (D) Statistical data of cell cycle distribution. *p < 0.05, **p < 0.01, ***p < 0.001 versus control. Data are presented as the mean ± SEM of three independent experiments. (E) Heatmap of the mRNA expression of critical differentially expressed genes using RNA-seq data derived from two samples (treated and untreated PG-4c, p < 0.05). (F) GSEA of migration-related gene sets indicating suppression of motility pathways following PG-4c treatment. [file Image1.tif]
